# Marked Decrease in CA 19-9 Level Belies Rapidly Progressive Lymphangitic Carcinomatosis in a Case of Metastatic Pancreatic Cancer

**DOI:** 10.1089/pancan.2020.0015

**Published:** 2020-11-16

**Authors:** Daniel A. King, Gino Pineda, Iny Jhun, George Fisher

**Affiliations:** ^1^ Division of Oncology, Department of Medicine, Stanford University, Stanford, California, USA.; ^2^ Department of Pathology, Stanford University, Stanford, California, USA.

**Keywords:** CA 19-9, FOLFIRNIOX, biomarker limitations, lymphangitic carcinomatosis, pancreatic cancer, pancreatic ductal adenocarcinoma

## Abstract

***Background:*** The CA 19-9 tumor marker is commonly used alongside imaging to trend chemotherapy response in patients with pancreatic ductal adenocarcinoma.

***Presentation:*** We describe an unusual clinical case of metastatic pancreatic cancer who achieved a marked decline in CA 19-9 but paradoxically developed widespread pulmonary lymphangitic carcinomatosis leading to rapid clinical decline and death.

***Conclusions:*** This case highlights the limitations of using the CA 19-9 tumor marker in isolation.

## Case Report

**FIG. 1.**
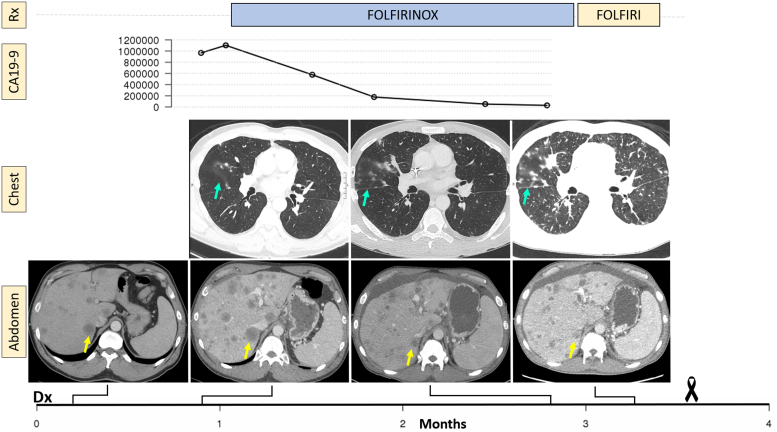
Timeline showing the treatments received, the CA 19-9 levels measured, and representative images from the CT scans of pulmonary and hepatic disease involvement. At diagnosis, the patient was found to have widespread hepatic metastases (bottom left). At the pretreatment scan, the index lesion (yellow arrow) had enlarged, but then decreased in size, along with a markedly improved CA 19-9 level. However, the patient had development and progression of pulmonary opacities (blue arrow). CT, computed tomography.

A 53-year-old previously healthy man presented in April 2020 with progressive epigastric pain and fatigue for several weeks. Computed tomography (CT) scan of the chest, abdomen, and pelvis (CAP) showed a 4.3 × 4.2 × 4.0 cm mass in the tail of the pancreas, peripancreatic lymphadenopathy, and metastatic spread to the liver (Fig. 1). His pretreatment CA 19-9 tumor marker level was markedly elevated at 1.1 million units per mL (U/mL). Biopsy of the pancreatic tail mass confirmed pancreatic ductal adenocarcinoma, with intact DNA mismatch repair proteins MLH1, MSH2, MSH6, and PMS2. Targeted genetic profiling identified a *KRAS* G12V mutation and no additional mutations. His performance status was excellent and the care team selected modified 5-FU, irinotecan, and oxaliplatin (mFOLFIRINOX) for treatment. In May 2020, a pretreatment baseline CT of the CAP showed disease progression since the initial scan, with worsening hepatic metastases, enlarging lymphadenopathy, new sites of osseous metastases, and new pulmonary nodules. The scan also identified bilateral pulmonary emboli for which anticoagulant therapy was initiated.

The patient began mFOLFIRINOX therapy. The CA 19-9 trend was suggestive of excellent response, with levels decreasing exponentially at 2, 4, and 6 weeks of therapy. The patient experienced severe nausea on treatment and oxaliplatin and irinotecan were dose reduced. He additionally developed shortness of breath around the time of his third cycle. He completed four cycles of treatment and follow-up CT of the CAP in mid-July was suggestive of mostly positive response: there was improvement in the hepatic burden, for example, the segment 6 lesion that was previously 3.2 × 3.1 cm in size decreased to 2.1 × 1.7 cm, and the right hepatic dome lesions that was 3.7 × 2.7 cm decreased to 2.8 × 2.3 cm; improvement in lymphadenopathy, such as an aortocaval node originally 4.4 × 3.2 cm decreased to 2.5 × 1.7 cm; however, there were new bone lesions, increasing ascites, and the lungs showed “redemonstration of multiple bilateral pulmonary nodules with associated nodular interlobular septal thickening, overall subtle but increased from prior exam.” At the time of this scan, his CA 19-9 level reached a nadir of 28,612 U/mL.

Given the dramatic decline in CA 19-9 levels as well as the improvement in pancreatic and hepatic disease burden, the development of lung nodules and septal thickening was surprising and the patient was referred to pulmonology to advise on the differential of the lung findings, including progression of pulmonary metastatic disease, failure of anticoagulant therapy, drug toxicity, or infection. Given further functional decline with fatigue and nausea, oxaliplatin was stopped and he received FOLFIRI for the fifth cycle of treatment. He then met with pulmonology, who performed a bronchoscopic biopsy of two hilar lymph nodes, which demonstrated metastatic adenocarcinoma compatible with a pancreatic primary. At this time, his dyspnea worsened, and his ascites worsened, requiring paracentesis, with cytology showing malignant cells. Owing to his rapidly declining clinical condition, the patient made the decision to not move forward with further chemotherapy. He transitioned to hospice and died a week later.

**FIG. 2.**
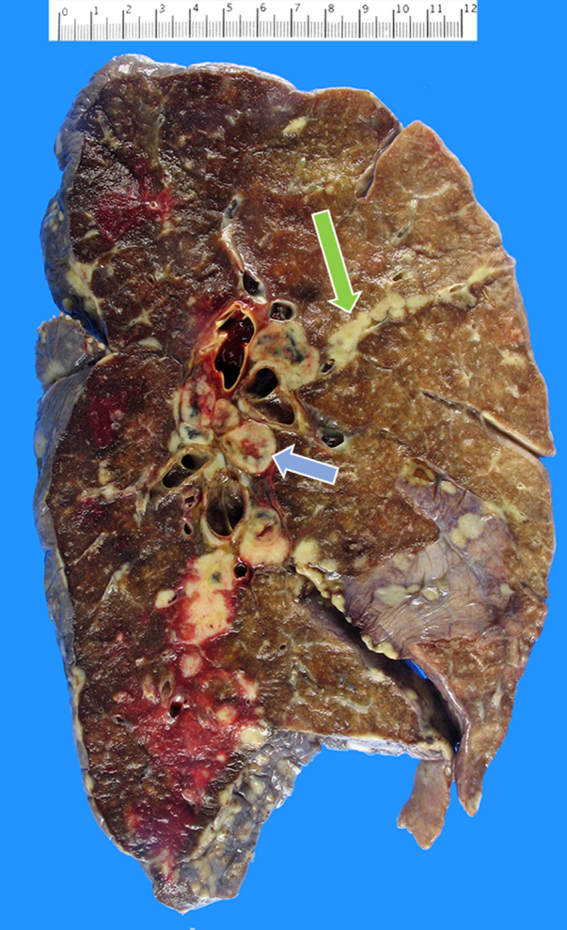
Left lung showing diffuse metastatic nodular deposits and extensive hilar lymphadenopathy (blue arrow), with tumor growth beyond the lymphatic vessels resulting in lymphangitic spread with intersegmental infiltration (green arrow).

**FIG. 3.**
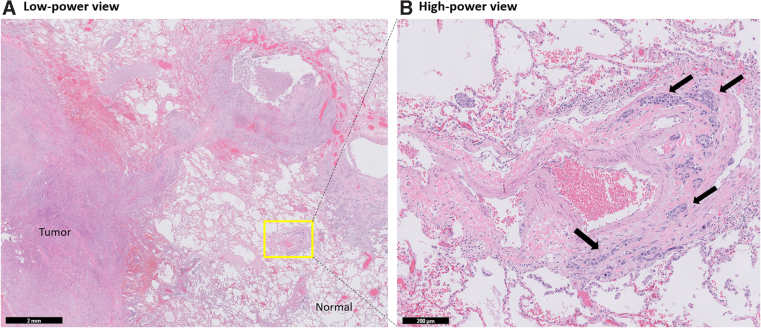
Microscopic evaluation of lung demonstrated **(A)** multiple small nodular tumor deposits (<1 cm) throughout the lung parenchyma and **(B)** lymphangitic spread of tumor cells adjacent to a large pulmonary vessel (black arrows). **(B)** is the magnification of the region (yellow box) in **(A)**.

Autopsy showed widely metastatic adenocarcinoma and a 10-cm primary mass at the head and neck of the pancreas that was densely adherent to nearby vascular and biliary structures, leading to occlusion of the common bile duct. The lungs demonstrated diffuse bilateral involvement by nodular (most <1 cm) and lymphangitic tumor deposits (Fig. 2). Microscopic examination showed aggregates of tumor cells expanding the periarterial lymphatic vessels (Fig. 3), consistent with pulmonary lymphangitic carcinomatosis (PLC). There was no evidence of macroscopic or microscopic pulmonary emboli in either lung. The liver showed widespread nodular tumor deposits involving 40% of the hepatic parenchyma with only a minor proportion of scattered tumor cells showing treatment effect.

## Discussion

This case is notable for a dramatic discordance between CA 19-9 levels, which suggested marked response, and clinical disease progression, observed during chemotherapeutic treatment of metastatic pancreatic cancer.

Metastatic pancreatic ductal adenocarcinoma is a noncurable disease with a median survival of <1 year from the time of diagnosis.^1^ Monitoring of disease response to chemotherapy is crucial to distinguish patients who are achieving disease control from those who are not to avoid harmful continuation of an ineffective and also often toxic therapy. Pancreatic cancer is among the few cancer types with a blood-based tumor marker, CA 19-9, in wide use for cancer monitoring, for patients whose tumors produce this antigen.^2^ Decrease in CA 19-9 level has generally been used as an indicator of response to chemotherapy, such as gemcitabine and FOLFIRINOX, and is associated with better patient outcomes^3–6^; however, this is somewhat controversial, as noted in a recent pancreatic cancer PDAC study wherein initial pretreatment CA 19-9 *level*, but not change during treatment, was prognostic,^7^ suggesting that CA 19-9 levels may not be a universally applicable biomarker to assess response.

Guidelines recommend assessment of treatment response using serial imaging in addition to measurement of the blood glycoprotein CA 19-9 (Category 2B),^8^ but not by CA 19-9 alone.^9^ Limitations in both measurement strategies exist. Regarding imaging, given the extent of desmoplastic stroma in pancreatic tissue, “radiographic findings may appear stable despite dramatic falls in CA 19-9.”^8^ There are also limitations to using CA 19-9, as (1) it is not produced by ∼10% of the population,^10^ (2) it can be elevated from nonmalignant processes that affect the biliary system reaching levels into the thousands,^6,11,12^ and (3) most studies^13,14^ but not all^7^ demonstrate that change in CA 19-9 levels during chemotherapy for patients with metastatic disease correlates with treatment benefit.

The patient already described developed shortness of breath, and autopsy confirmed widespread development of PLC. Pulmonary tumor emboli and PLC are malignant pulmonary embolization forms that are considered as end-stage manifestations of malignancy. This patient developed widespread PLC in the absence of evidence of tumor emboli. PLC represents tumor in the lung lymphatic channels and is associated with invasion to the interstitium resulting in thickened septa,^15,16^ which was the likely source of pulmonary metastasis in our patient. The prevalence of PLC in pancreatic cancer is not known, but is rare compared with primary cancers of the lung, breast, or stomach (in order of descending frequency).^15–19^ The poor prognosis associated with lymphangitic carcinomatosis is reported in other primary tumor types.^20,21^ This has promoted emphasis on early diagnosis of this type of spread, as well as how to best detect and diagnose lymphangitic carcinomatosis more conclusively, by biopsy^21^ or a high-resolution CT for patients for whom a biopsy is not feasible.^22^ By our review of the literature, no study to date has reported CA 19-9 levels in a patient with PLC.

We had entertained whether the CA 19-9 measurement, given its substantial magnitude at diagnosis (967,402 U/mL), might have been artifactual. However, several observations argued against this: (1) repeat measurement performed 1 week later, also before starting treatment, remained considerably elevated (1,103,315 U/mL); (2) the CA 19-9 value was below the maximum reporting limit of our laboratory's assay; (3) in fact, far higher CA 19-9 levels have been reported in the literature in patients with pancreas cancer, such as one report of 19.5 million U/mL in which through serial dilution and an interference assay was shown to be reliable.^23^ Therefore, we had several reasons to believe the validity of the CA 19-9 levels observed pretreatment.

The apparent lack of association between our patient's CA 19-9 biomarker and progression in the lung confounded interpretation of disease response to treatment. As noted previously, our patient's pretreatment CA 19-9 levels were substantially elevated and declined drastically once chemotherapy was initiated. Had CA 19-9 remained high or continued to elevate after starting FOLFIRINOX, it may have helped interpret the mixed response on scan as progression, triggering change of therapy.

Several hypotheses may explain the discordance observed between CA 19-9 response and progression of disease. It is possible that he truly had a mixed response, whereby there was indeed a reduction in burden of disease in the liver and lymph nodes while separately small deposits of scattered tumor and lymphangitic spread—both difficult to observe radiologically—were occurring in the lungs. However, microscopic examination of the liver deposits demonstrated only a minor treatment effect. It is conceivable that the production of CA 19-9 by tumor cells is affected by organ site, with variable production, secretion, or systemic translocation of CA 19-9 by tissue. Whether there is a reduction of CA 19-9 through these mechanisms in the lungs may merit additional investigation.

This case also suggests that an additional measurement, orthogonal to imaging and CA 19-9, namely circulating tumor DNA (ctDNA), may be useful for monitoring disease progression. Indeed, repeated mutant *KRAS* ctDNA measurements during follow-up are being explored as one potential strategy, and appeared to be superior to protein-based tumor markers in detecting progressive disease.^24^ A potential advantage of this approach is that *KRAS*, which is a clonal driver of pancreas cancer, is expected to be present and expressed by all tumor cells, whereas CA 19-9 expression or release from tumor cells may be more variable.

This patient's unusual combination of pancreatic adenocarcinoma and PLC illustrates shortcomings in current practices for monitoring treatment response, particularly when patients experience an uncommon path of disease progression. This cautions against over interpretation of CA 19-9 levels especially when patients present with a rare and poor prognostic course, such as lymphangitic carcinomatosis.

## Abbreviations Used

CAP: chest, abdomen, and pelvis
CT: computed tomography
ctDNA: circulating tumor DNA
mFOLFIRINOX: modified 5-FU, irinotecan, and oxaliplatin
PLC: pulmonary lymphangitic carcinomatosis
